# Oscillatory phase transition induced structural extension during iron oxide reduction

**DOI:** 10.1016/j.fmre.2023.10.023

**Published:** 2024-01-05

**Authors:** Haoyang Fu, Qingze Chen, Benzhi Min, Shuzhou Li, Xiaodong Chen, Lan Ling

**Affiliations:** a State Key Laboratory for Pollution Control and Resource Reuse, College of Environmental Science and Engineering, Tongji University, Shanghai 200092, China; b School of Materials Science and Engineering, Nanyang Technological University, Singapore 639798, Singapore; c CAS Key Laboratory of Mineralogy and Metallogeny/Guangdong Provincial Key Laboratory of Mineral Physics and Materials, Guangzhou Institute of Geochemistry, Chinese Academy of Sciences (CAS), Guangzhou 510640, China

**Keywords:** Oxide reduction, In-situ TEM, Catalysis, Epitaxial nanoislands, Nonclassical reduction

## Abstract

Probing the molecular-level redox behavior and mechanism of oxides is essential to developing innovative applications in different fields such as catalysis, but remains a challenge for the scientific community. Using in-situ transmission electron microscopy, here we provide an overall reduction view of single-crystalline α-Fe_2_O_3_ with different characteristics from the conventional wisdom of oxide reduction. Specifically, the formation of epitaxial nanoislands with concomitant oscillatory phase transitions (α-Fe_2_O_3_→defective γ-Fe_2_O_3_→α-Fe_2_O_3_) at the subsurface is observed during reduction. The dynamic equilibrium of lattice oxygen at the surface and the limited oxygen replenishment from the deep layer drive the α-Fe_2_O_3_→defective γ-Fe_2_O_3_ transformation in the subsurface, while the polymorphic transition (defective γ-Fe_2_O_3_→α-Fe_2_O_3_) spontaneously occurs under heating conditions. Such oscillatory phase transition is accompanied by the release of asymmetric stress, inducing the extension of epitaxial nanoislands. Our work highlights the complexity of reduction by providing an integral picture of oxide reduction, which contributes to the understanding of the site evolution of oxide-based catalysts in their working state.

## Graphical abstract

**Figure:**
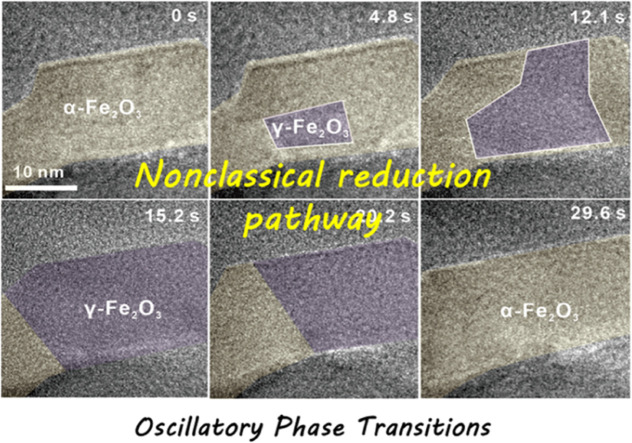

## 1 Introduction

Transition metal oxides are among the most abundant geological materials with a huge impact on celestial bodies, like Earth, Mars, and Moon [1]. Meanwhile, they are evidenced to be a class of highly active catalysts for various catalytic oxidation/reduction reactions including CO_2_ reduction [2,3], methanol synthesis [4,5], and oxygen reduction reaction [6,7], due to their unique physicochemical features. Understanding the structural characteristics of metal oxides at the atomic scale is of great interest to uncover the catalytic mechanisms and tune the functional properties. However, the metal oxides may undergo oxidation/reduction during the catalysis process, leading to changes in their electronic states and surface topology. Such local morphological and chemical changes would significantly impact the activity and selectivity of the catalysts, making it challenging to understand the structure-activity relationships and predict the behavior of the catalyst in a particular reaction [8], [9], [10]. Therefore, an atomic-level understanding of the redox mechanism of metal oxides is necessary for exploiting high-performance catalysts.

Recently, several surface-sensitive spectroscopic methods including X‑ray scattering and the temperature-programmed reduction have been performed to study the oxide redox behavior [11], [12], [13]. Although these spectroscopic techniques are helpful to understanding the reduction theory, the influence of micro factors on the redox process cannot be overlooked as the aforementioned techniques are statistical averaging tools, which renders the observations unrepresentative of the oxides in their working state [14,15]. As a result, questions remained regarding time and spatial mismatches between the dynamic changes and the spectroscopies used. In this respect, constructing a comprehensive understanding of oxide redox behavior requires capturing the evolution of local microstructure with sufficient spatial and temporal resolution. Unfortunately, there is limited knowledge about the operando structural and physiochemical properties of the oxides as well as how they evolve during redox reactions, and the picture of the entire oxide redox is still far from clear—even direct visualization of the dynamics process within an individual particle is still rarely reported to date [16,17].

Recent developments in scanning/transmission electron microscopy (S/TEM) in imaging using a gas environmental cell offers the opportunity to obtain the details of oxide redox dynamically at the atomic level. In this study, we focused on the visualization of the reduction dynamics of the typical transition metal oxide, α-Fe_2_O_3_. With the aid of the in-situ TEM characterization, an overall reduction process of α-Fe_2_O_3_ with different characteristics from the previously reported reduction view is observed. Unlike the classical nucleation/contracting sphere reduction, the epitaxial nanoislands can be formed and extended by the local cyclic reduction of α-Fe_2_O_3_ followed by the reoxidation (α-Fe_2_O_3_→defective γ-Fe_2_O_3_→α-Fe_2_O_3_) process in our case. The continuous release of asymmetric stress during oscillatory phase transition drives the outward extension of the epitaxial region to form the FeO_x_ chains. Our results highlight the diversity of reduction pathways, which has broader significance for understanding the active sites of transition metal oxides in the working state and provides a valuable guide for the further design of high-performance catalysts with improved activity and selectivity.

## 2 Experimental methods

### 2.1 Synthetic procedure of single-crystalline α-Fe_2_O_3_

The single-crystalline α-Fe_2_O_3_ was synthesized via the hydrothermal method. Briefly, 0.273 g of iron trichloride (FeCl_3_·6H_2_O, Aladdin Industrial Co., China) was dissolved in 10 mL of ethanol with an addition of 3 mL of water. Subsequently, 0.8 g of sodium acetate (CH_3_COONa, Aladdin Industrial Co., China) was added under stirring (500 rpm). Then, the mixture was stirred for 1 h and sealed in a Teflon-lined autoclave (25 mL), heated at 180 °C for 12 h. The obtained products were washed 3 times with distilled water and dried for characterization.

### 2.2 In situ environmental study of α-Fe_2_O_3_ reduction

In situ experiments were performed using a commercial Protochips gas-cell TEM holder inside the FEI Titan G^2^ 60–300 spherical aberration-corrected TEM at an acceleration voltage of 200 kV. The gas-cell holder is composed of two chips: the top one with several electron-transparent windows made of SiN_x_ membrane, and the bottom one featuring a micro-patterned heater integrated with the same SiN_x_ membrane windows. With this gas-cell holder, it can elevate the temperature of the sample from 20 °C to 1300 °C while maintaining a gas pressure of 10^5^ bar. Procedures for calibration were implemented to determine the upper limit of electron beam dosage that the system could withstand without sustaining damage. When performing in situ reduction experiments, the high-purity H_2_ (Messer, purity > 99.999%) gas was inserted into a gas reaction cell using a DENSsolutions gas supply system (Delft, Netherlands). The gas was introduced at a flow rate of ≈ 0.1 mL·min^−1^ (pH_2_ = 10^5^ bar), and the temperature was raised from 25 °C to 650 °C in 5 min with a heating rate of 1 °C/s. The in-situ high-angle annular darkfield STEM images were captured in STEM mode, and the electron energy loss spectroscopy (EELS) data were collected with a Gatan Tridiem spectrometer.

It should be noted that electron beam effects cannot be avoided in in-situ TEM experiments. To eliminate the electron-beam effect in our reduction experiments, we subjected the sample to electron beam irradiation at *T* = 650 °C for 2 h. Additionally, we adjusted the imaging conditions in one area and then moved to a neighboring area to minimize the electron beam effect during HRTEM imaging. This approach allowed us to reduce the repetition of electron beam exposure and avoid the potential impact of electron beam damage on our samples.

### 2.3 Simulation method

Large-scale Atomic/Molecular Massively Parallel Simulator (LAMMPS) was used to conduct the molecular dynamics (MD) simulation processes in this study [18]. An orthogonal box with parameters $a=b=c=464.82\;\text{Å},\alpha =\beta =\gamma =90^{\circ }$, was constructed as the simulation box, which contains a sphere of Fe_2_O_3_ crystal composed of 216-unit cells (unit cell parameters: $a=b=5.035\;\text{Å},c=13.72\;\text{Å},\alpha =\beta =90^{\circ },\gamma =120^{\circ }$) with a radius of 25 Å and 1,500 H_2_ molecules (Supporting Information Fig. S1). The reactive force field [19], which is a reliable MD force field to reproduce the iron redox reactions, was applied to the simulation system. The whole system was energy minimized and then heated at 1,500 K with the Nose-Hoover thermostat for 1,000 picoseconds. The Visual Molecular Dynamics and Open Visualization Tool were used to display and animate molecular dynamics simulation trajectories.

To describe the behavior of oxygen migration from the inner layer to the surface layer of iron oxide, a function named Oxygen Hopping was defined as Eq. (1).

(1) $$H\left(t\right)=\sum_{i=1}^{i=n}H_{i}\left(t-\Delta t\right)+H_{i}\left(\Delta t\right)$$

where t is the running time of MD simulation, Δt is the time interval of two adjacent trajectory frames. In this study, H(∆t) of each oxygen atom could have three values as shown in Eq. (2).

(2) $$H\left(\Delta t\right)=\left\{ \begin{array}{c} 0,\;no\;hop\\ 1,\;hops\;front\\ -1,\;hops\;back \end{array}\right.$$

It is comprehensible that $H_{i}\left(\Delta t\right)$ of oxygen atom i would be 0 when it stays in place where there is no change of its coordination environment. $H_{i}\left(\Delta t\right)=1$ means oxygen atom i moves to replace a new oxygen vacancy where it has different coordinated Fe atoms from last step (t−Δt). Similarly, $H_{i}\left(\Delta t\right)$ can be defined as −1 when atom i moves to the location at step t−2Δt. To quantitatively analyze the oxygen migration in different layers, the iron oxide sphere was divided into 11 layers and each layer was named “layer 1″ through “layer 11″ from the surface to the centroid. The thickness of each layer was 3.4 $\overset{\circ }{A}$ as the O—O pair separation distance has the first minimum at 3.4 $\overset{\circ }{A}$.

The density functional theory (DFT) calculations of this study are all calculated by the Vienna Ab initio Simulation Package program. The calculations were carried out using the projector augmented wave (PAW) method with a cutoff energy of 600 eV. The electronic exchange-correlation energy is determined by generalized gradient approximation with the revised Perdew-Burke-Ernzerhof (PBEsol) functional. PBEsol functional was introduced to improve the equilibrium properties of solids. Valence-core interactions were described using PAW pseudopotentials. The Brillouin zone sampling was done using the (3 × 3 × 1) Monkhorst-Pack grids for surface and Gamma for the structure. The structure optimization iterations are set to be 2 × 10^−6^ eV and 0.002 eV/Å respective to the energy and force convergence. The formation energy of oxygen vacancy E_p_ was calculated using the following equation:

(3) $$E_{p}=E_{t}-E_{r}+\frac{N_{0}}{2}E_{O}$$

where, E_t_ is the total energy of the Fe-O system containing N_o_ number of oxygen vacancies, E_0_ is the energy of an isolated oxygen molecule, and E_r_ is the energy of the structure without the oxygen vacancies.

## 3 Results and discussion

### 3.1 Visualization of reduction dynamics

**Fig. 1:**
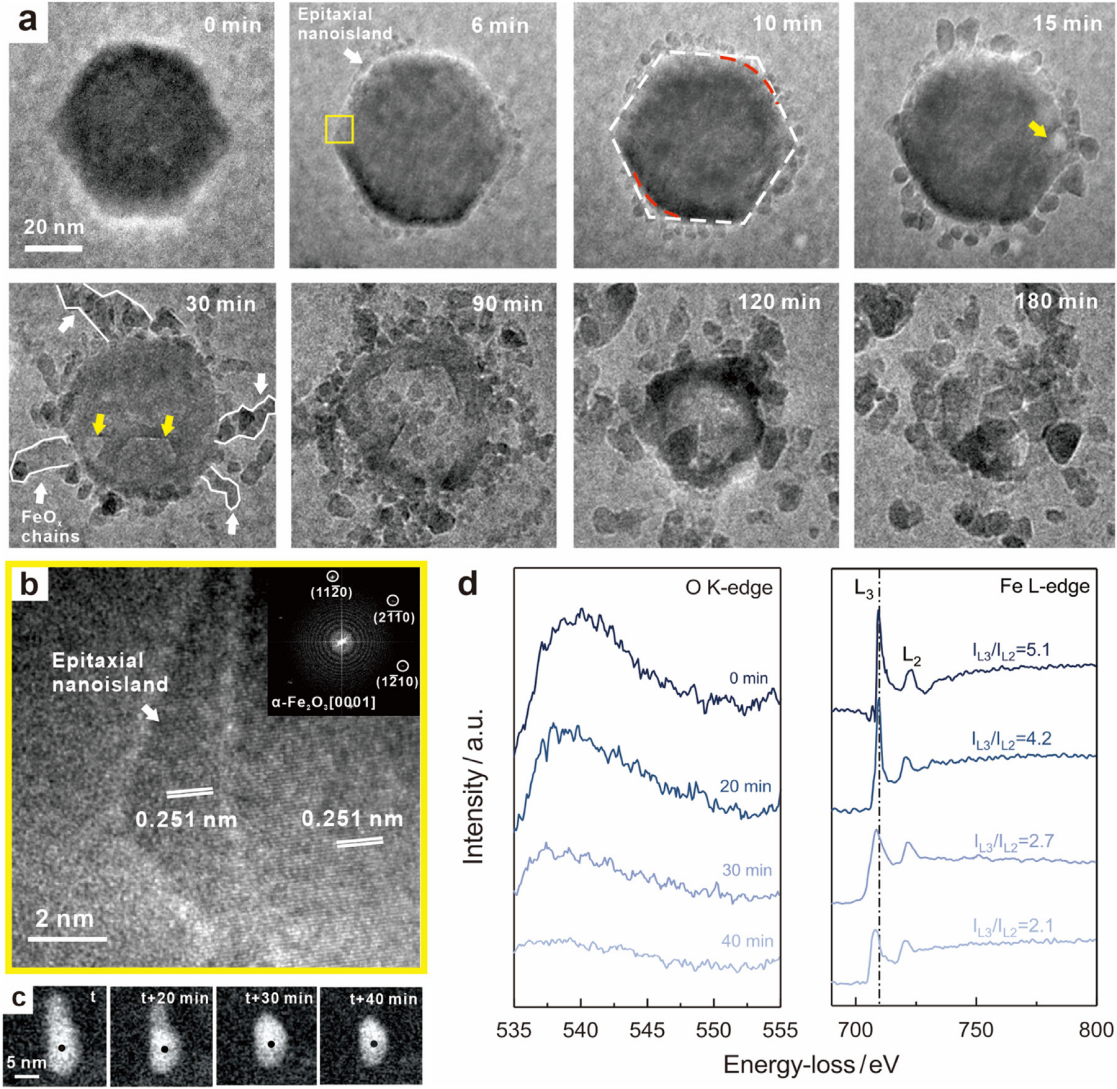
**The nonclassical reduction pathway of α-Fe_2_O_3_.** (a) Time-resolved TEM images of the reduction of individual α-Fe_2_O_3_ nanoparticle at *T* = 650 °C and pH_2_ = 10^5^ bar show a different picture from the classical nucleation/contracting sphere reduction theory. (b) The HRTEM image of the epitaxial nanoisland/parent oxide interface are marked by the yellow line in (a), and the inset is the corresponding FFT pattern, showing the epitaxial nanoisland in the same phase as the parent oxide. (c) The time-resolved STEM images of the reduction of separated FeO_x_ chains, revealing the rapid reduction of FeO_x_ chains. (d) EELS spectra of O K-edge and Fe L-edge marked by circles in (c) recorded at different reduction times.

Here the well-defined single-crystalline α-Fe_2_O_3_ with hexahedral morphologies was selected to probe its reduction dynamics. Using the single crystal is helpful to deriving the fundamental trends of phase transformation during reduction [20]. Detailed synthetic procedure and characterization of α-Fe_2_O_3_ are described in the Methods and Supporting Information Fig. S2. For in-situ observations of α-Fe_2_O_3_ reduction, the α-Fe_2_O_3_ are dispersed on a holey carbon film supported on a gas-cell TEM holder. The α-Fe_2_O_3_ are reduced at 650 °C with 101 kPa H_2_ flowing into the gas cell within the aberration-corrected TEM. Note that the heating rate is 2 °C/s, and there is no morphological change during such rapid heating process. Fig. 1 shows the real-time TEM images (snapshots from Movie S1 in Supplementary Information) recorded at different stages of α-Fe_2_O_3_ reduction. It can be seen that the first around 6 min of continuous H_2_ flow through the cell is an incubation period, during which the α-Fe_2_O_3_ particle remains relatively stable without significant structural changes (Fig. S3). After 10 min of reduction, the strain-relaxed structure at the corners of α-Fe_2_O_3_ is observed, followed by the formation of some epitaxial nanoislands. The initial phase of epitaxial nanoislands has α-Fe_2_O_3_ structure as revealed by HRTEM images and fast Fourier transform (FFT) pattern, identical to the parent phase (Fig. 1b). Further reduction progress (15 min) leads to a loss of shape anisotropy (particle becomes round; Red dashed line) of the parent phase along with the concomitantly forming of epitaxial nanoislands at the edges. As the reduction proceeded, several pits appear at the edge of the parent phase as indicated by the yellow arrow, and those pits gradually propagate and expand toward the center of the parent phase with time (30 min). At the same time, the epitaxial nanoislands extend outward and then form the FeO_x_ chain (Fig. S4). Continuous reaction leads to the gradual segregation of chains from the parent phase and then the extension of the FeO_x_ chains terminated (90 min). The separated FeO_x_ chains rapidly integrate and coalesce into several nanoparticles in the subsequent 30 min. Time-dependent EELS spectra further monitored the gradual reduction process of newly formed nanoparticles during a time elapse of 40 min (Fig. 1c-d), as evidenced by the progressive fading characteristics of O K-edge and the decrease of white-line intensity ratio (L_3_/L_2_) in the Fe L_2,3_-edge from 5.1 to 2.1 [21]. Such nanoparticles eventually become quasi-spherical and the lattice fringes of the final phase observed in HRTEM are identified as metallic Fe. For the parent phase, a drastic decrease in particle size was found, followed by disintegration and reduction into smaller metallic Fe particles (Fig. S5). The fragmentation upon redox systems is common because of the huge discrepancy in structure indexed with lattice parameters between oxidizing and reducing species [17,22].

Typically, the reduction of oxide is described with the nucleation model or contracting sphere model [16,23,24]. For the nucleation model, the reduced phase is nucleated firstly at the edge of parent oxides. As reduction continues, these nuclei expand and coalesce, ultimately leading to the complete reduction of the parent oxide, with the reduced phase replacing the oxide entirely. In the contracting sphere model, a uniform and continuous reduced phase layer rapidly forms around the oxide particle, and the reduced-phase shell contracts inward as reduction proceeds. Although phenomenological models have been effective in describing oxide reduction, the reduction behavior observed in this work shows a different scenario from the classical reduction theory. Rather than forming a reduced phase on the outermost surface, our observations reveal that the α-Fe_2_O_3_ reduction starts with the formation of epitaxial nanoislands. Moreover, the expansion of epitaxial nanoislands can be clearly and consecutively observed, which indicates that reduction is an autocatalytic process in the sense that growth proceeds spontaneously at the growth front once the nanoislands have built up. Such a process is triggered when the H_2_ molecule is preferentially adsorbed on the growth-front of the epitaxial nanoisland as the geometries for this adlayer are fully relaxed [25]. By monitoring the oxygen content of the epitaxial region and the formed pit in the parent phase, we find that the oxygen content in the pit region tends to decrease over time, while the oxygen content of the epitaxial region only shows small fluctuations before the epitaxial region detached from the parent phase (Supporting Information Fig. S6). This means that the lattice oxygen in the epitaxial region can be sufficiently replenished even if it is preferentially attacked by external H_2_. Considering that the FeO_x_ chains are rapidly reduced after deviating from the parent phase, prior to which the detachment of chains can be viewed as triggered by the insufficient oxygen supply from the parent phase to the chains, we speculate that the formed epitaxial regions are the channel for the outward oxygen diffusion from the bulk of α-Fe_2_O_3_ during reduction. In this regard, a detailed understanding of the expansion process of epitaxial regions is necessary to verifying our assumption and gaining deep insight into this nonclassical reduction behavior.

### 3.2 Local redox cycle in the epitaxial region

**Fig. 2:**
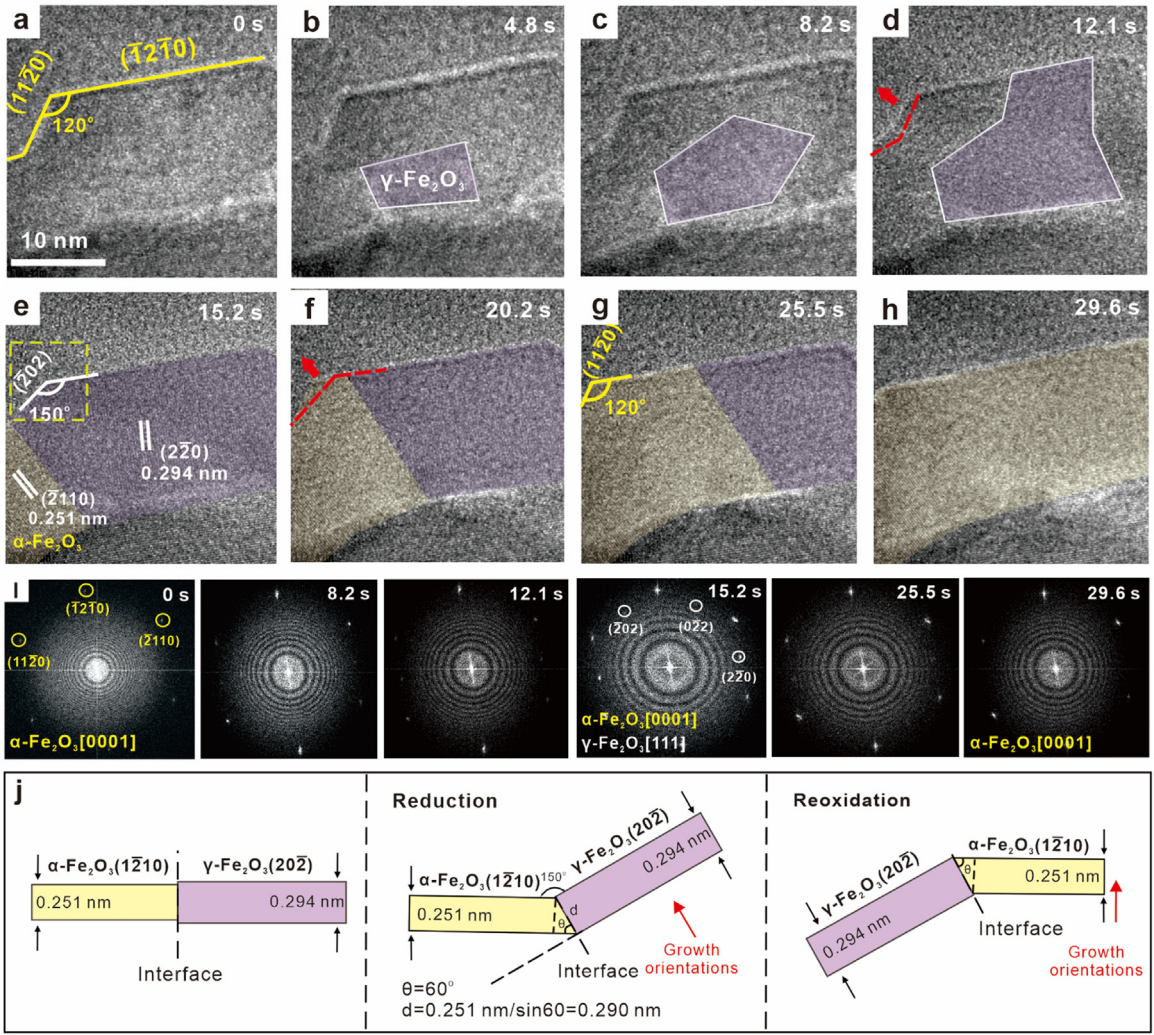
**Atomic-scale visualization of oscillatory phase transition.** (a) Time-resolved HRTEM images of α-Fe_2_O_3_ reduction in the epitaxial region at *T* = 650 °C and pH_2_ = 10^5^ bar. The purple and yellow areas show the regions of defective γ-Fe_2_O_3_ and α-Fe_2_O_3_ phases, respectively, revealing the reduction and reoxidation process of α-Fe_2_O_3_ in the epitaxial region over time. The red dashed lines in d and f are the traces of the position and configuration of the surface at 9 s and 15 s, respectively, which show the expansion of epitaxial nanoislands induced by the oscillatory phase transition. (i) The corresponding FFTs of each HRTEM image. (j) Schematics of the interface of α-Fe_2_O_3_($1\bar{2}10$) plane and the γ-Fe_2_O_3_($\bar{2}02$) plane during reduction and reoxidation process, revealing that the slantwise growth mode has a lower interfacial lattice mismatch compared to the perpendicular growth mode (a TEM image of higher resolution is provided in Fig. S7).

Fig. 2 displays an in-situ HRTEM observation of the extension of epitaxial nanoislands. The initial FFT pattern of the selected epitaxial nanoisland displays one set of diffraction spot, corresponding to α-Fe_2_O_3_ phase, and the epitaxial nanoisland shows the well-defined and atomically clean ($\bar{1}$2$\bar{1}$0) and ($11\bar{2}$0) facets (Fig. 2a). After H_2_ exposure for 4.8 s, the small pit starts to form at the subsurface (a depth of ≈ 5 nm) of epitaxial nanoisland (Fig. 2b). This pit then quickly enlarged with the ongoing reduction, accompanied by the gradually growing of epitaxial nanoisland (Fig. 2c, d). FFT pattern of the pit region shows a new set of diffraction spots corresponding to γ-Fe_2_O_3_ (or Fe_3_O_4_) phase with 〈111〉 zone axis (Fig. 2i), indicating that the formation of a new reduced phase (i.e., γ-Fe_2_O_3_ or Fe_3_O_4_) in the subsurface. Because both γ-Fe_2_O_3_ and Fe_3_O_4_ have similar cubic structures with almost identical lattice parameters (γ-Fe_2_O_3_: P4_1_32, *a* = *b* = *c* = 0.835 nm; Fe_3_O_4_: Fd$\bar{3}$m, *a* = *b* = *c* = 0.839 nm) [26,27], those two phases cannot be differentiated on the basis of the diffractograms. However, the presence of octahedral Fe vacancy ordering in γ-Fe_2_O_3_ sets it apart from Fe_3_O_4_ [28]. In γ-Fe_2_O_3_, one-sixth of the octahedral Fe sites are empty, while in Fe_3_O_4_, all octahedral Fe sites are occupied [26], which leads to important differences in the EELS spectra, reflecting the local arrangement of the Fe atoms. It can be found that the Fe L-edge energy positions of the pit region (marked with the red circle) are located quite close to that of γ-Fe_2_O_3_ reference, which confirms the γ-Fe_2_O_3_ structure in the pit region (Supporting Information Fig. S8). By fitting the measured spectrum to the standard FeO and α-Fe_2_O_3_ spectra, the relative content of Fe^2+^ and Fe^3+^ is quantified as 12.3% and 87.7%, respectively. The generation of Fe^2+^ species is the result of a countercurrent flow of electrons triggered by the lattice oxygen loss [9]. Those confirm that the formation of the pit is induced by the transformation from α-Fe_2_O_3_ to the defective γ-Fe_2_O_3_ (with oxygen vacancies). Note that here the limited oxygen vacancies in the pit region are not sufficient to present the extra superlattice spots associated with the oxygen-vacancy-ordering structure in the FFT image (Fig. 2i). Similar phase transformation pathway is also reported in the previous studies, which reveals that the defective γ-Fe_2_O_3_ is the first reduced phase during α-Fe_2_O_3_ reduction [8,29]. In addition, the transformation of α-Fe_2_O_3_ to defective γ-Fe_2_O_3_ along the α-Fe_2_O_3_(0001)/γ-Fe_2_O_3_(111) interface does not require much change in atomic arrangement since these two planes are close-packed with a hexagonal in-plane symmetry at the interface, and thus the topotactic transition with the crystallographic orientation relationship of α-Fe_2_O_3_[0001]//γ-Fe_2_O_3_<111> is identified.

The extension of epitaxial nanoisland and transformation of α-Fe_2_O_3_ to defective γ-Fe_2_O_3_ are temporarily terminated when the corner angle (marked by the yellow dashed lines) changes from 120 to 150 ± 2° at the reduction time of 15 s or so. Here the propagation of the atomic steps flattens the surface and forms the γ-Fe_2_O_3_ ($\bar{2}02$) surface (Fig. 2e). As illustrated schematically in Fig. 2j, if the γ-Fe_2_O_3_ phase grows parallelly on the α-Fe_2_O_3_($1\bar{2}10$) plane, the interfacial lattice misfit between α-Fe_2_O_3_ and γ-Fe_2_O_3_ reaches 15% [(0.294 − 0.251)/0.294 ≈ 15.0%], resulting in large lattice strain. Instead, the interfacial lattice misfit is reduced to 1.4% [(0.294 − 0.290)/0.294 ≈ 1.4%] when the angle between α-Fe_2_O_3_($1\bar{2}10$) and γ-Fe_2_O_3_($\bar{2}02$) becomes 150° (Fig. 2j). To achieve the least interfacial lattice misfits and lower grain boundary energy, the growth of γ-Fe_2_O_3_ at an inclined angle (i.e., 30°) on the epitaxial nanoisland is more favorable than parallel growth with α-Fe_2_O_3_ ($1\bar{2}10$) plane. Therefore, it is comprehensible that the preferential growth orientations determined by interfacial lattice misfits allow γ-Fe_2_O_3_ to grow linearly along the [0001] direction. The growth of γ-Fe_2_O_3_ ($\bar{2}02$) plane causes the corner angle of the epitaxial nanoisland to deviate by 30° (i.e., 120° to 150°). More interestingly and importantly, the defective γ-Fe_2_O_3_ is then reoxidized back to α- Fe_2_O_3_ over time. After ∼4 s, the α-Fe_2_O_3_ phase in the bottom left of Fig. 2d gradually grows and penetrates deeper into the γ-Fe_2_O_3_ region while the essential grain boundary maintains (Fig. 2f-g). The FFT pattern reveals that the epitaxial nanoisland completely oxidized to α-Fe_2_O_3_ after ∼30 s of reduction (Fig. 2i). On the other hand, further propagation of the epitaxial nanoisland is observed, with the corner angle changing again from 150° to 120° (Fig. 2h). By allowing the difference between α-Fe_2_O_3_ ($1\bar{2}10$) and γ-Fe_2_O_3_ ($\bar{2}02$) by 1.4% at an angle of 150°, the lattice misfit induces the α-Fe_2_O_3_ to grow along the [0001] direction for strain relaxation (Fig. 2j). This process facilitates the formation of ($1\bar{2}10$)-type facet of α-Fe_2_O_3_, which has a corner angle of 120° with ($11\bar{2}0$) facet. Further observation of the epitaxial region showed that such oscillatory phase transition (α-Fe_2_O_3_→defective γ-Fe_2_O_3_→α-Fe_2_O_3_) frequently occur during reduction. In a lower magnification view of the reduction dynamics, we can find that the phase oscillation in the epitaxial region has a sequential order, which starts from the subsurface and then gradually occurs in the deeper layer (Supporting Information Fig. S9 and Movie S4). The end of one redox cycle in the outer surface is accompanied by the cyclic occurrence in the deeper region. As revealed in Supporting Information Fig. S9c, d, when the pit near the expansion surface becomes smaller, one pit emerges in the region of ≈ 8 nm below the reaction front. One full cycle of the reduction and reoxidation takes 10–35 s. More cases of the similar oscillatory phase transition are provided in Supporting Information Fig. S10.

While similar oscillatory transitions have also been found for the case of copper (Cu→Cu_2_O→Cu) in the model reaction of hydrogen oxidation by Willinger et al. [25] and Huang et al. [30], as revealed in this work, it is notable that the oscillatory phase transition still exists even in the reducing atmosphere (i.e., H_2_). During such a local redox-cycle process, the asymmetric stress is continually released by the outward extension of epitaxial nanoislands, resulting in the formation of FeO_x_ chains. The growth orientation of nanoislands induced by oscillatory phase transition is anisotropic and depends on the angle at which the two phases of α-Fe_2_O_3_ and γ-Fe_2_O_3_ reach lattice matching. Results obtained from the single-crystalline α-Fe_2_O_3_ with dominantly exposed different facets are mutually consistent, providing robust evidence for the oscillatory phase transition-induced nanoisland extension (Figs. S11, 12). Note that the effect of vacuum annealing and electron beam on the oscillatory phase transition can be neglected in our system (Fig. S13 and Movie S3).

### 3.3 Simulation modeling and analysis of subsurface redox cycle

**Fig. 3:**
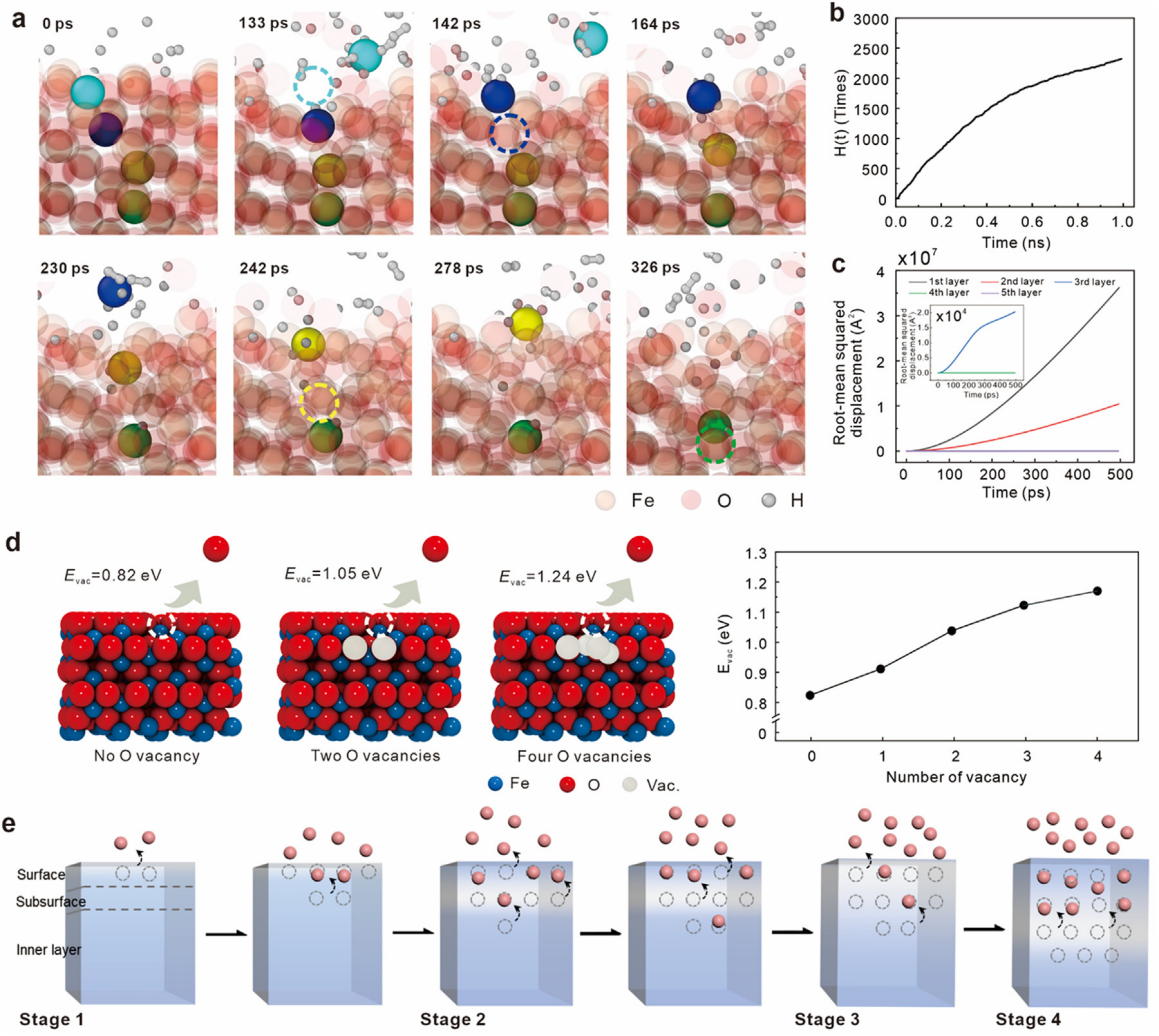
**Simulation modeling of subsurface redox cycle.** (a) Snapshots from MD simulations shows the oxygen migration behavior. (b) Root-mean square displacement of oxygen atoms in different layers. (c) Oxygen hopping as function of simulation time. (d) Surface oxygen vacancy formation energy as a function of the concentration of oxygen vacancies in α-Fe_2_O_3_. (e) Schematics of the subsurface redox cycle process.

Further in-situ TEM experiments reveal that the local redox cycle is closely related to the reduction condition that is observable within a temperature range of 600 to 800 °C. No observable oscillatory phase transition-induced extension of epitaxial nanoislands was found when the reduction temperature decreases to 500 °C, where the reduction behavior can be well explained using the classical interface model (Fig. S14). At higher reduction temperatures (1,000 °C), the sintering and splitting behavior becomes prominent, which may be caused by the rapid phase transition inducing substantial lattice stresses (Fig. S15). The apparent discrepancy may be the result of the diffusional mass transfer that controls the practical reduction pathway. The redox cycle can be considered as being driven by the change in the relative rates of release and replenishment of lattice oxygen in the subsurface. The reduction from α-Fe_2_O_3_ into defective γ-Fe_2_O_3_ (with oxygen vacancies) in the subsurface region implies that here the replenishment of lattice oxygen is overwhelmed by outward diffusion. To further understand such a process at an atomic level, we conducted MD simulations using a reactive force field which is developed specifically for iron-oxyhydroxide systems and has been proven to be suitable for iron redox reactions [19]. To mimic the experimental conditions, a spherical Fe_2_O_3_ nanoparticle is put at the center of cubic simulation box under an H_2_ atmosphere as the lattice oxygen atoms on the surface are released, the formed oxygen vacancies are quickly replenished by the oxygen atoms in the second layer at 142 ps (Fig. 3a; Movie S5). After that, the newly formed oxygen vacancies in the second layer are further supplemented by the third layer of lattice oxygen, while the oxygen migrating from the second layer to the surface has entered the H_2_ atmosphere in the form of a H_2_O molecule (230 ps). Finally, the oxygen in the third layer migrated to the surface and desorbed from the surface after 326 ps. At this time, the fourth layer of oxygen appeared to migrate upward. The oxygen migration process shown above indicated that the surface oxygen vacancy can be quickly replenished, and oxygen vacancy replenishment time gradually increases from the surface to the inside layers. The first-, second-, and third-layer oxygen vacancy replenishment time is 9, 88, > 100 ps, respectively. Such oxygen migration phenomenon is indeed revealing a non-vehicular oxygen hopping process during reduction, which is attributed to the presence of oxygen vacancy defect in the system. Hopping is defined as the movement of an oxygen atom to a new oxygen vacancy, which is coordinated with Fe atoms that is completely different from the previous position. Additionally, the oxygen atom must not return to its previous position in the subsequent step [31,32]. We find that the oxygen hopping took place during the whole simulation time and the oxygen hopping rate (the slope of hopping vs time curve) decreased with time, which is shown in Fig. 3b. In addition, the mean square displacement (MSD) of oxygen atoms for every layer is calculated (Fig. 3c). Obviously, the diffusion of oxygen atoms is basically weaker in the inner layer. The analysis above shows that it is more difficult for the inner layer oxygen vacancies to get oxygen supplements compared to that of the surface. Specifically, when the oxygen of the surface layer is desorbed from the surface first leaving oxygen vacancy, the lattice oxygen in the second layer can quickly replenish the surface oxygen vacancy while the replenish speed of inner layer oxygen is much slower.

On the other hand, once oxygen atoms of the inner layer migrated to the surface, the subsequent desorption of migrated oxygen into the H_2_ atmosphere requires more energy than the surface oxygen desorption at the beginning. It can be seen that the desorption time of the first-layer oxygen is 61 ps, and the time of oxygen desorption after migration from the second and third layers to the first layer increase to 88 and 100 ps, respectively (counted from the time of one H proton capture by lattice oxygen) (Movie S5). Such a phenomenon can be visually displayed by counting the change in the number of oxygen atoms at each layer during simulation (Movie S6). We find that the surface oxygen slightly decreased at the beginning and then remained basically constant with time, whereas the oxygens in the second and third layers decreased rapidly. This implies that the inner layer is unable to keep up with the influx of oxygen vacancies as the reaction proceeds, while the rate of lattice oxygen loss is higher than the rate of oxygen supply at the beginning and then reached a dynamic equilibrium of oxygen loss and replenishment at the surface. The lattice oxygen loss would be difficult if the energy required for oxygen vacancy formation based on a higher energy cost. By evaluating the formation energies of surface oxygen vacancies in the presence of oxygen vacancies in the inner layer, we can gain insight into why the desorption ability of surface oxygen that migrates from the inner layer decreases with time. The DFT calculations are utilized to investigate the energetics related to the formation of surface oxygen vacancy at various numbers of oxygen vacancies in inner layers. Fig. 3d shows that the formation energy of surface oxygen vacancies gradually increases from 0.82 to 1.24 eV when the number of oxygen vacancies increases from 1 to 4, which suggests that the more oxygen vacancies in inner layers, the less favorable the detachment of surface oxygen. This behavior of enhanced energy cost induced by internal vacancies slows down the surface oxygen loss and therefore actively affects the outward migration of lattice oxygen in inner layers. We know that the phase reduction is the result of the accumulation of overpopulated oxygen vacancies [33,34]. Above simulations have rationalized that the observed transformation from α-Fe_2_O_3_ to defective γ-Fe_2_O_3_ is induced by an accumulation of oxygen vacancies in the subsurface region. Kinetically, the higher concentration of oxygen vacancies at the subsurface can be achieved through two stages: (1) the oxygen deficiency at the surface exerts a driving force for the rapid outward diffusion of inner lattice oxygen, which, together with the limited oxygen replenishment at the subsurface, leads to the inward diffusion of oxygen vacancies; (2) the accumulation of oxygen vacancies in the subsurface slows down the surface oxygen loss, keeping the surface oxygen vacancies at a low concentration (Fig. 3e). It is worth noting that similar behavior of inward diffusion of oxygen vacancies during CuO reduction has also been recently found by Zhou's group [35]. On the other hand, the subsequent γ-Fe_2_O_3_ phase growth from the inside out further points to the continuous lattice oxygen loss from the surface layer (Stage 3; Fig. 3e), which also provides evidence that the accumulation of oxygen vacancies starts in the interior.

**Fig. 4:**
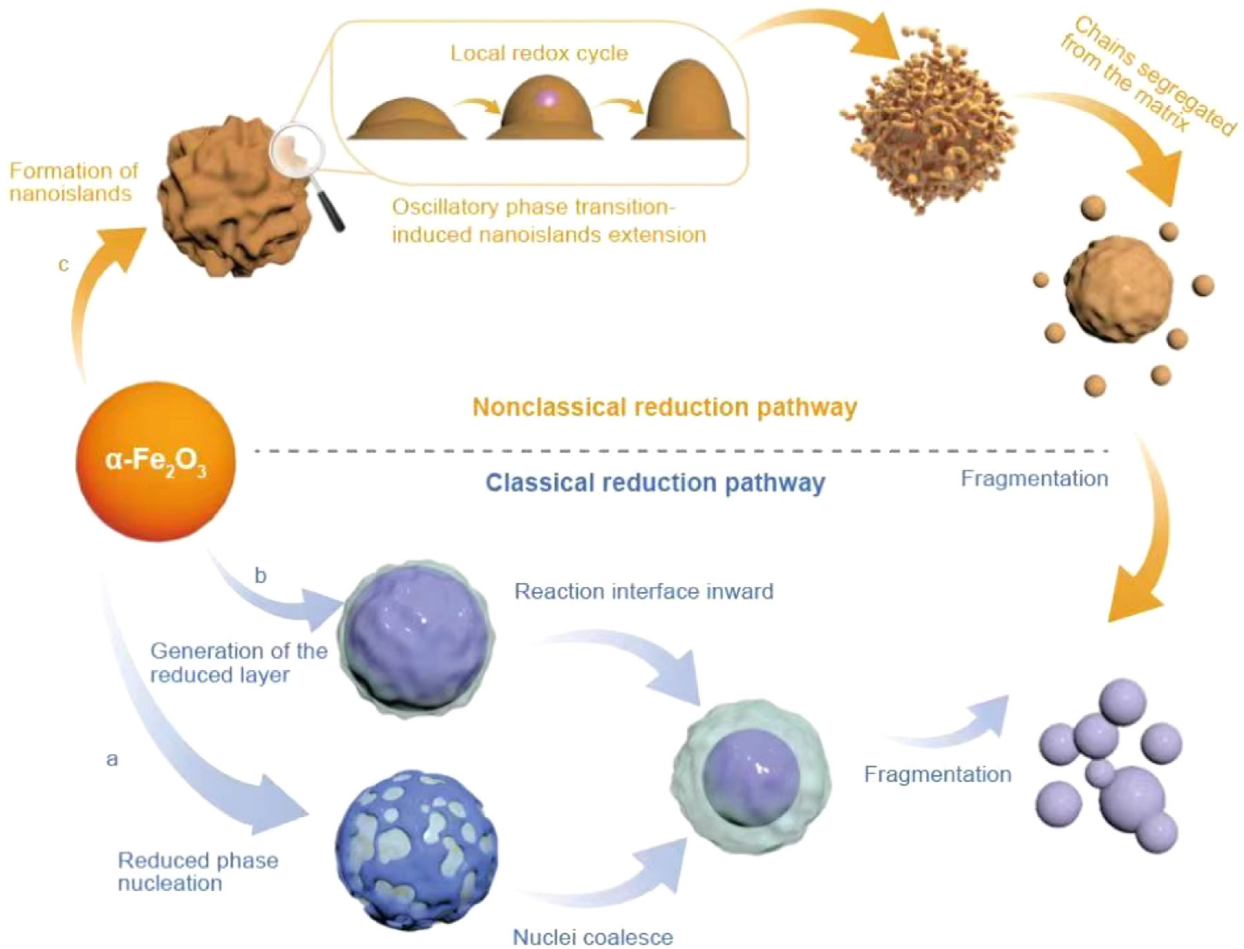
**Schematic comparison of the reduction models.** (a) The nucleation model. The reduction process begins with the nucleation of small metal clusters on the surface of the oxide. These clusters then grow by the addition of atoms, eventually forming a continuous metal layer that spreads across the oxide surface. (b) The contracting sphere model. The reduction process occurs through the shrinking of a contracting sphere of reduced metal around each oxide particle. (c) Nonclassical reduction pathway. The epitaxial nanoislands are formed and extended during the α-Fe_2_O_3_ reduction by the local cyclic reduction of α-Fe_2_O_3_ followed by reoxidation (α-Fe_2_O_3_→ defective γ-Fe_2_O_3_ →α-Fe_2_O_3_). The continuous release of asymmetric stress during oscillatory phase transition drives the outward extension of epitaxial region and formation of the FeO_x_ chains.

It is noted that the α-Fe_2_O_3_→defective γ-Fe_2_O_3_ transformation is accompanied by the strain release that has been described above, in which the external thermal energy (heat treatment) further results in the thermodynamical unstablity of the remaining newly formed γ-Fe_2_O_3_ [36,37]. Being the metastable phase with relatively low surface energy (0.71 ± 0.13 J·m^2–^), γ-Fe_2_O_3_ prefers to transform to α-Fe_2_O_3_ phase that has surface energy in the higher side (1.9 ± 0.3 J·m^2–^) with thermal treatment [37]. Such a polymorphic transformation is crystallographically favorable and similar transition behavior has been observed by several studies on the aluminum oxide transition, where the crystallization of α-Al_2_O_3_ preferentially derives from the surface of γ-Al_2_O_3_ [38], [39], [40]. Considering that the reductive phase γ-Fe_2_O_3_ here is full of oxygen vacancies, filling the vacancies by surrounding oxygen becomes a prerequisite to the transformation from γ-Fe_2_O_3_ to α-Fe_2_O_3_. In the background of continuous oxygen out-diffusion from the bulk, it is therefore reasonable to believe that the defective γ-Fe_2_O_3_ phase acquires the oxygen atoms of α-Fe_2_O_3_ in the deeper layer to transform to α-Fe_2_O_3_, as the lattice oxygen of α-Fe_2_O_3_ in the deeper layer is the only source of oxygen supply. As oxygen atoms are taken from α-Fe_2_O_3_ in the deeper layers to fill vacancies in the defective γ-Fe_2_O_3_ phase, this process results in the loss of lattice oxygen from α-Fe_2_O_3_. This loss of oxygen and the accumulation of vacancies lead to the transition of α-Fe_2_O_3_ into defective γ-Fe_2_O_3_ in the deeper layers (Stage 4; Fig. 3e), as looking back to the reduction behavior in Movie S2. In essence, the difference in dynamics between oxygen replenishment and loss is the intrinsic motivation of the local redox cycle, which continuously drives the extension of epitaxial islands and then the nonclassical reduction behavior occurs. The nonclassical pathway for α-Fe_2_O_3_ reduction observed in this study and the classical pathway are schematically compared in Fig. 4. It is noted that the transformation of γ-Fe_2_O_3_ to α-Fe_2_O_3_ is a temperature-sensitive process, which generally starts at temperatures of up to ∼450 °C. When the reduction temperature decreases to 500 °C, the lattice oxygen in the surface of α-Fe_2_O_3_ would continuously release to form the defective γ-Fe_2_O_3_ phase, while the reoxidation of defective γ-Fe_2_O_3_ becomes slow and not capable of converting back to α-Fe_2_O_3_. As expected, the local oscillatory phase transition is no longer present during reduction. Instead, the reduction process follows a classical pathway as shown in Fig. S14.

## 4 Conclusion

To summarize, we reported an in-situ S/TEM study on the reduction of single-crystalline α-Fe_2_O_3_. A nonclassical reduction pathway that the formation and extension of epitaxial nanoislands induced by the oscillatory redox phase transitions (α- Fe_2_O_3_→defective γ-Fe_2_O_3_→α-Fe_2_O_3_) is observed. The dynamical reduction-reoxidation process starts in the subsurface of epitaxial nanoislands is a consequence of the rate difference between the lattice oxygen replenishment and loss. Together with quantitative computation, we reveal that the phase reduction of α-Fe_2_O_3_ into defective γ-Fe_2_O_3_ in the subsurface is attributed to the limited oxygen replenishment from the deeper layer and the dynamic equilibrium of oxygen capture and supply at the surface. The subsequent defective γ-Fe_2_O_3_ to α-Fe_2_O_3_ transformation is considered to be surface energy-driven polymorphic transformation under the high-temperature conditions. The continuous release of asymmetric stress during such oscillatory phase transition drives the outward extension of the epitaxial region. By combing laterally-resolving in-situ methods, our work highlight the diversity and complexity of reduction pathways and bridges the gap between the nanoscopic domains with molecular-level detail and the macroscopic regime of oxide reduction.

## Declaration of competing interest

The authors declare that they have no conflicts of interest in this work.

## Appendix Supplementary materials

Image, video 1 dummy alt text

Image, video 2 dummy alt text

Image, video 3 dummy alt text

Image, video 4 dummy alt text

Image, video 5 dummy alt text

Image, video 6 dummy alt text

Image, application 7
