# Supplementary material for: Oscillatory phase transition induced structural extension during iron oxide reduction
Source: Fundam Res. 2024 Jan 5;6(3):1459–67. doi: 10.1016/j.fmre.2023.10.023 (PMC13247456; doi:10.1016/j.fmre.2023.10.023)
Supplement: Supplementary file 7 [file mmc7.docx]

**Supporting Information for**

Oscillatory phase transition induced structural extension during iron oxide reduction

Haoyang Fu ^a,b #^, Qingze Chen ^c #^, Benzhi Min ^b^, Shuzhou Li ^b^, Xiaodong Chen ^b^, Lan Ling ^a *^

^a^ State Key Laboratory for Pollution Control and Resource Reuse, College of Environmental Science and Engineering, Tongji University, Shanghai 200092

^b^ School of Materials Science and Engineering, Nanyang Technological University, 50 Nanyang Avenue, Singapore, 639798

^c^ CAS Key Laboratory of Mineralogy and Metallogeny/Guangdong Provincial Key Laboratory of Mineral Physics and Materials, Guangzhou Institute of Geochemistry, Chinese Academy of Sciences (CAS), Guangzhou 510640

^#^These authors contributed equally to this work

* Lan Ling

**Email:**  Lanling@tongji.edu.cn

**This PDF file includes:**

Figures S1 to S14

**Other supporting materials for this manuscript include the following:**

Movies S1 to S6

**Movie S1** The reduction of the individual α-Fe_2_O_3_ nanoparticle at T= 650 ^o^C and pH_2_= 10^5^ bar.

**Movie S2** The reduction of α-Fe_2_O_3_ in the epitaxial region at T=650 ^o^C and pH_2_= 10^5^ bar.

**Movie S3** In situ TEM observation of the individual α-Fe_2_O_3_ nanoparticle at T= 650 ^o^C without the introduction of H_2_.

**Movie S4** A low magnification view of the oscillatory phase transition process.

**Movie S5** MD simulations of the oxygen migration behavior during reduction.

**Movie S6** The change in the number of oxygen atoms at each layer during MD simulations.


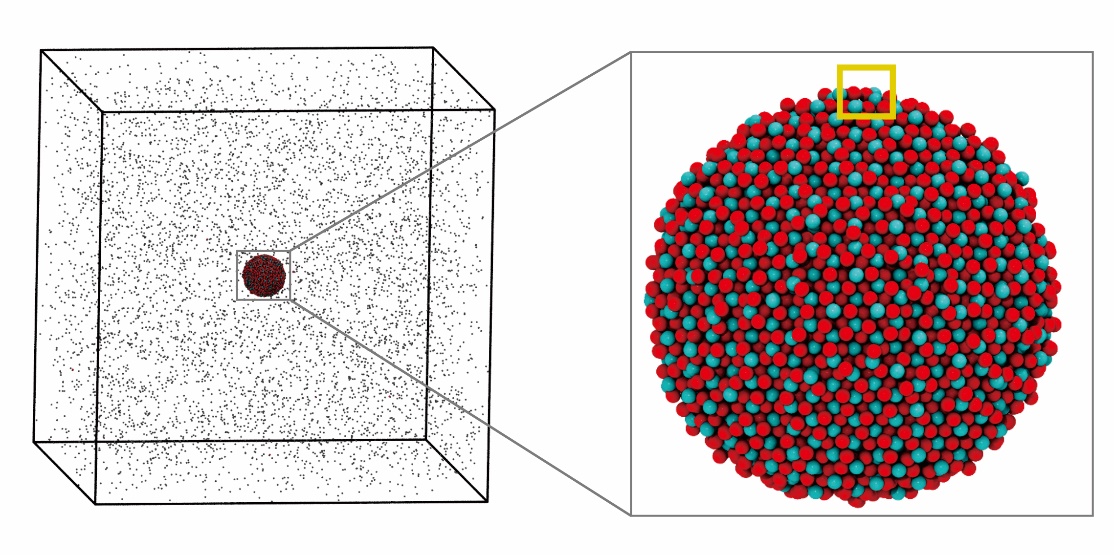


**Figure S1.** The constructed model for molecular dynamics simulation (The yellow rectangle corresponds to a specific view of Figure 3a).


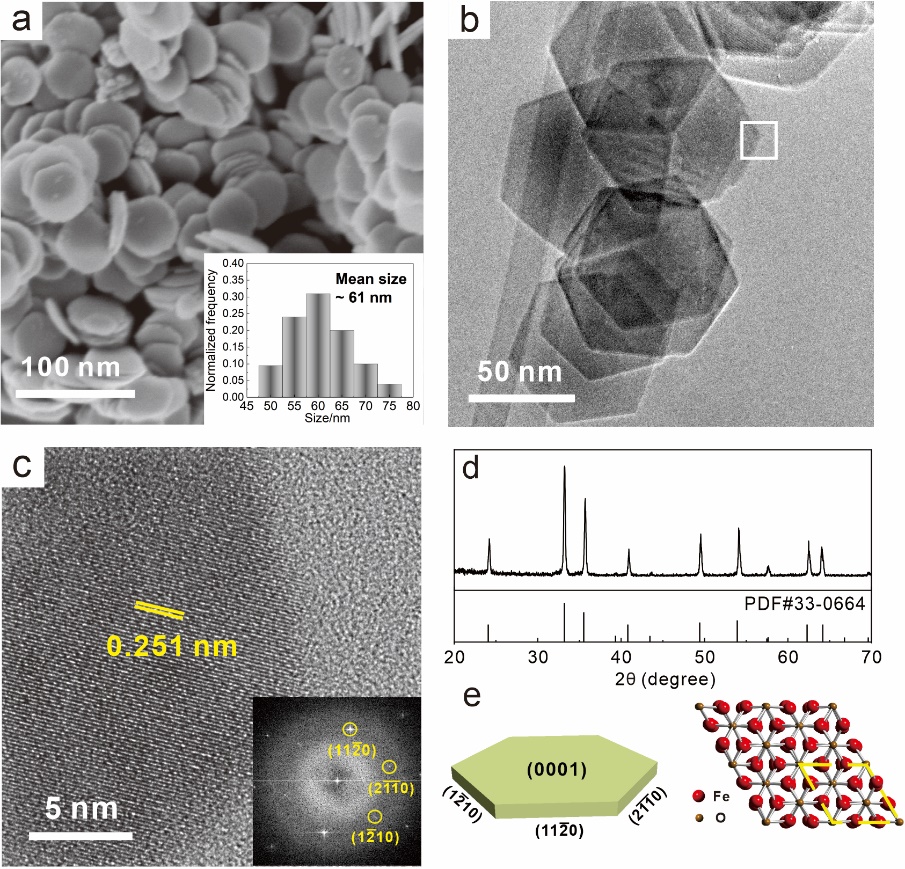


**Figure S2.** Representative morphologies and structure of α-Fe_2_O_3_. (a) SEM and (b) TEM image of α-Fe_2_O_3_, revealing α-Fe_2_O_3_ have hexahedral morphologies with an average width of 60 nm and thickness of 15 nm. Inset of (a) is the size distribution histograms of α-Fe_2_O_3_; (c) HRTEM image of α-Fe_2_O_3_, where the well-resolved lattice fringes with d-spacings of 0.251 nm corresponded to the distance between ($1\bar{2}10$) planes of α-Fe_2_O_3_ nanocrystals. Insert is the typical SAED pattern of α-Fe_2_O_3_; (d) XRD pattern of α-Fe_2_O_3_; (e) structure and schematic diagram of α-Fe_2_O_3_.


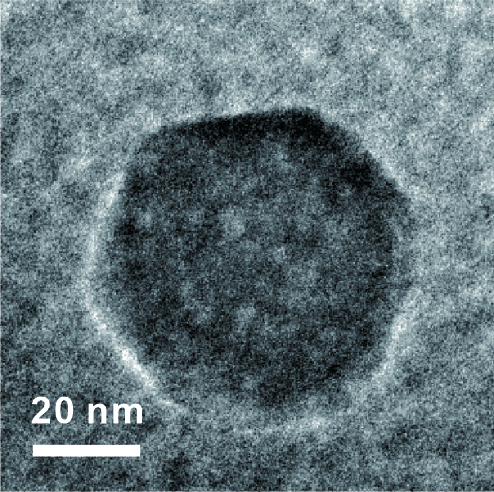


**Figure S3.** TEM image of the individual α-Fe_2_O_3_ nanoparticle after 5 min of reduction.


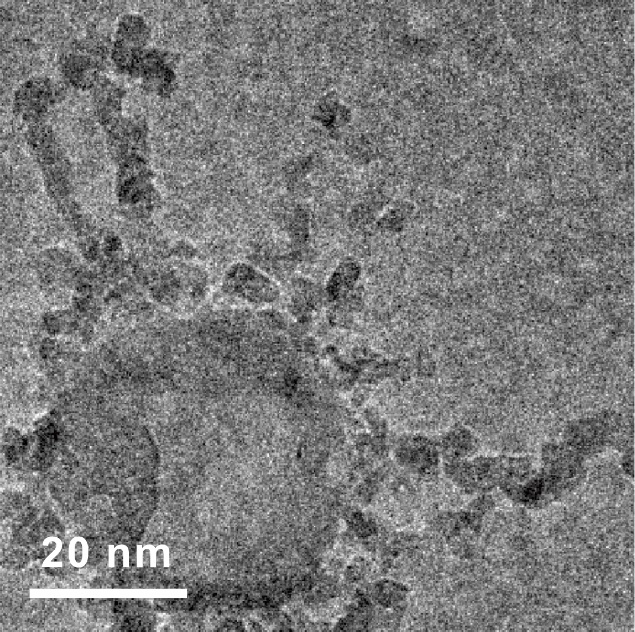


**Figure S4.** Low-resolution TEM image of the individual α-Fe_2_O_3_ nanoparticle after 45 min of reduction, revealing the outward extension of the epitaxial nanoislands followed by the formation of a chain-like structure.


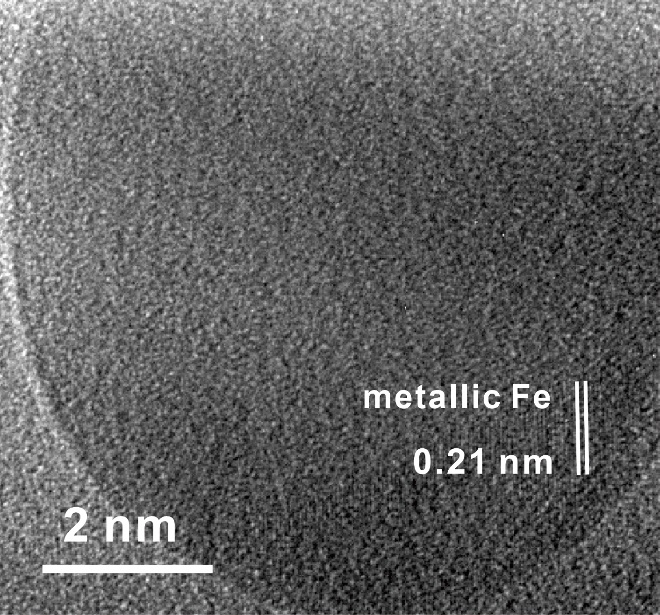


**Figure S5.** HRTEM image of the reduction phase disintegration from the parent oxide, revealing the eventually become the metallic Fe phase.


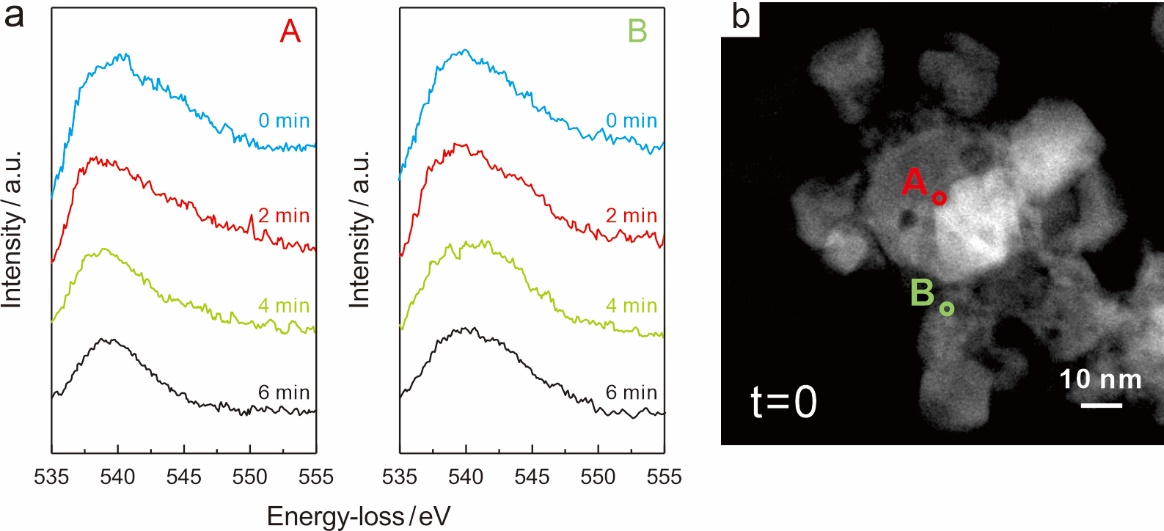


**Figure S6.** Time-sequence EELS spectra of O K-edge acquired at the pit region of the parent oxide (A point in (b)) and epitaxial region (B point in (b)), which reveals the oxygen content in the pit region tends to decrease rapidly with time, while the oxygen content in the epitaxial region shows only a slight decrease.

*
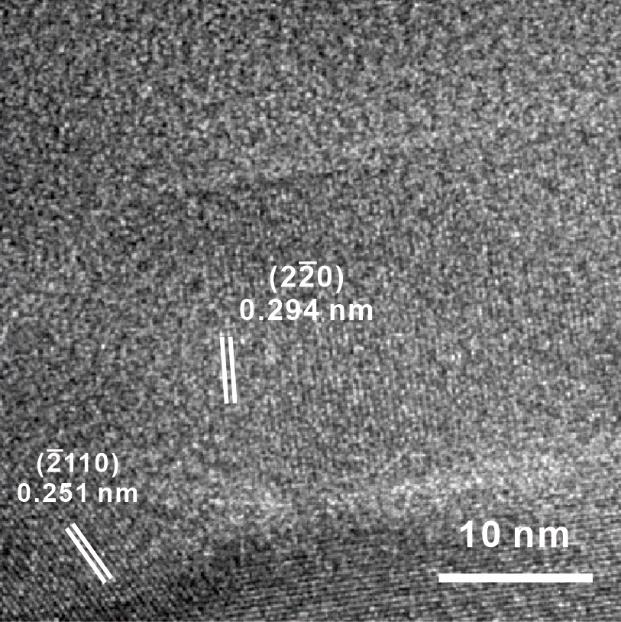
*

Figure S7 High-resolution TEM image of Fig. 2e


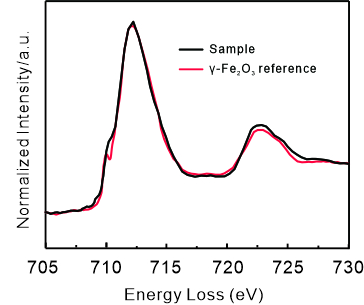


**Figure S8.** EELS spectra of Fe L-edge acquired at the pit region of the epitaxial nanoisland, revealing the phase in the pit region has the γ-Fe_2_O_3_ structure

**
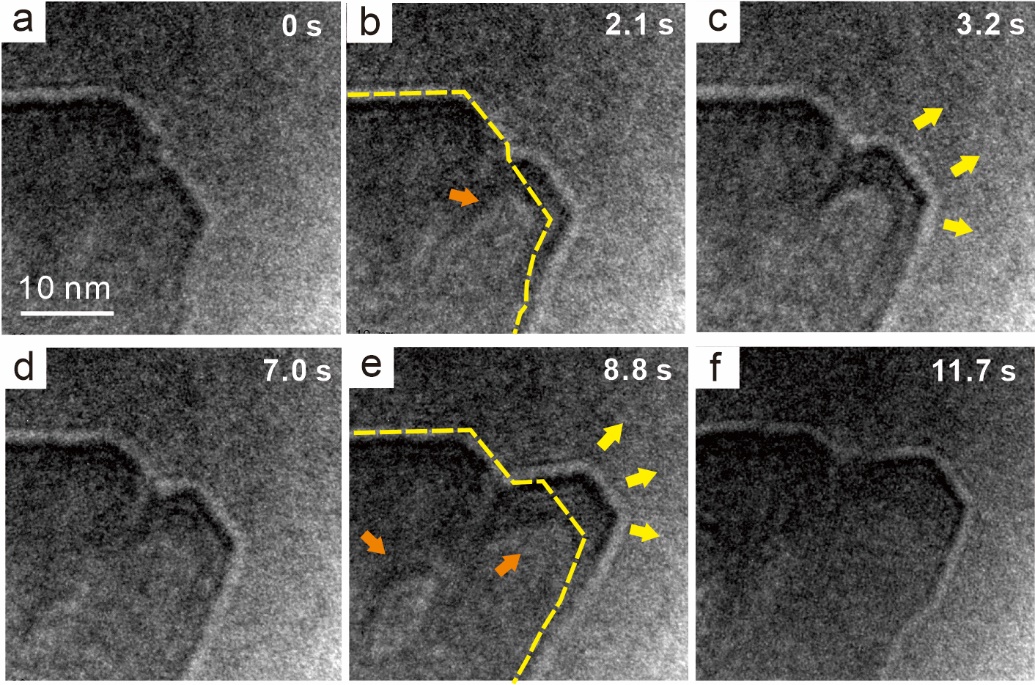
**

**Figure S9.** A low magnification view of the oscillatory phase transition, showing the phase oscillation in the epitaxial region has a sequential order, which starts from the subsurface region and then gradually occurs in the deeper layer.

Case 1:
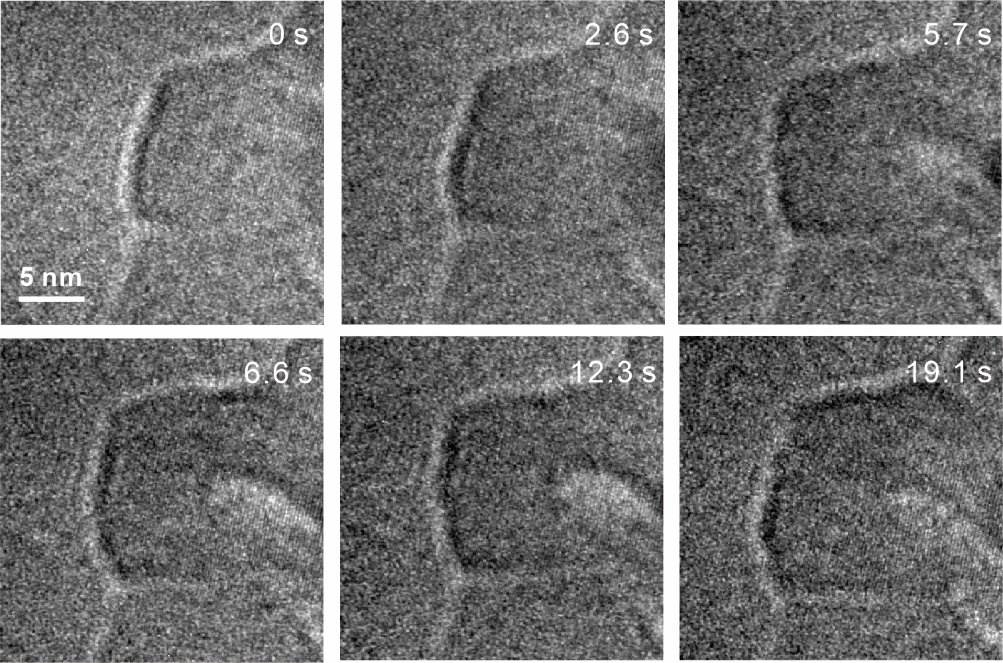


Case 2:
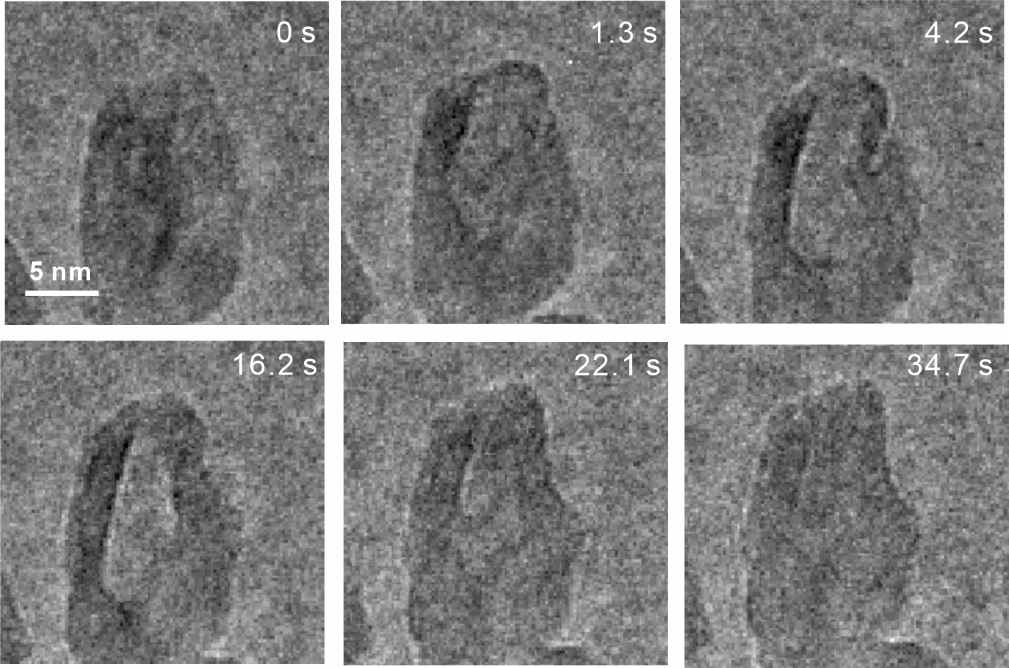


Case 3:
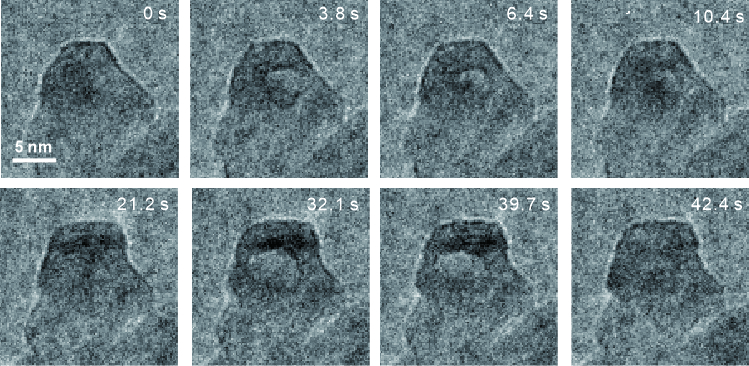


**Figure S10.** Three cases of oscillatory phase transition in the epitaxial nanoislands, which shows the full cycle of the reduction-reoxidation process takes 10-45 s.


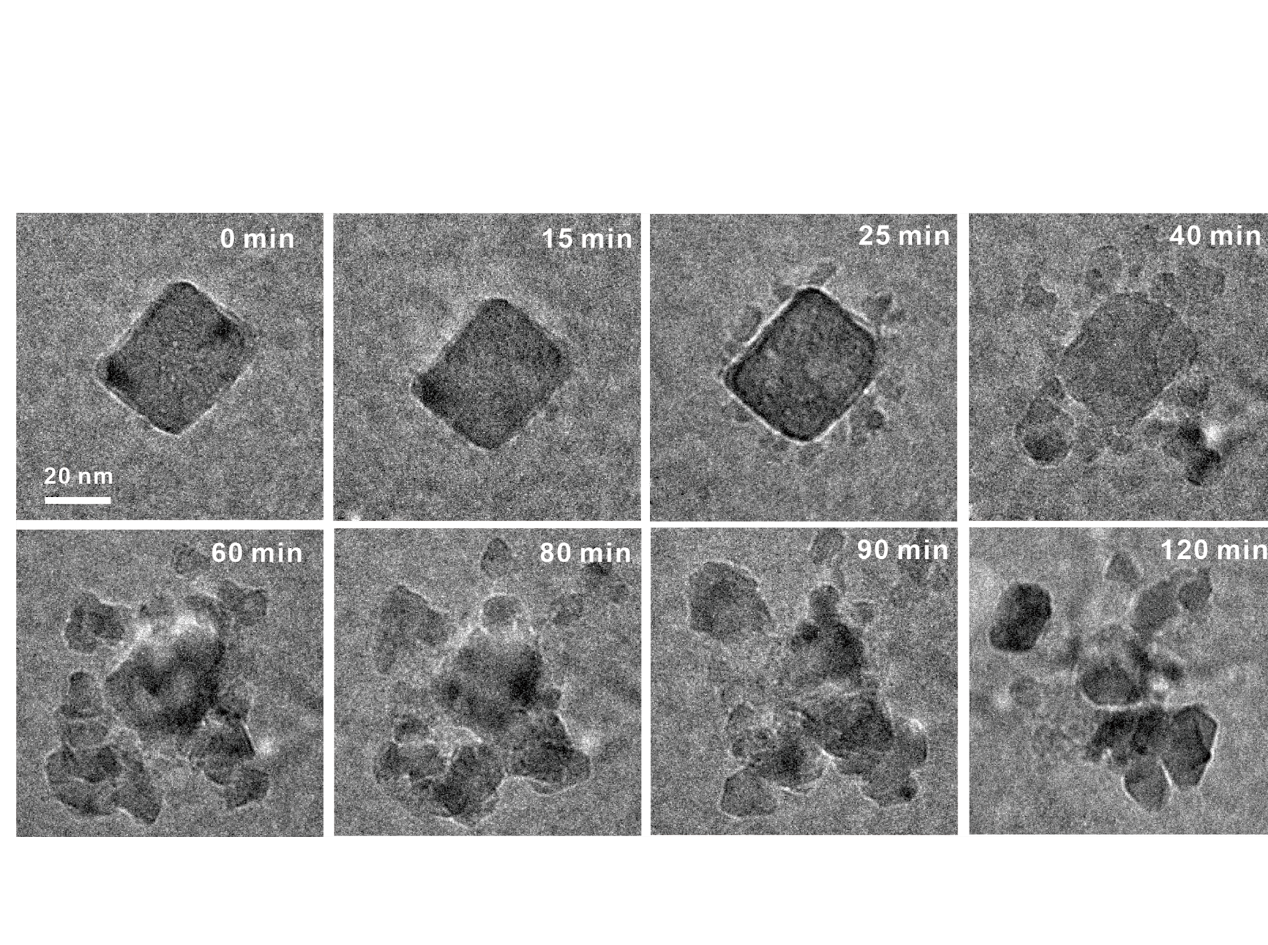


**Figure S11.** The nonclassical reduction behavior of α-Fe_2_O_3_ with dominantly exposed {110} facet.


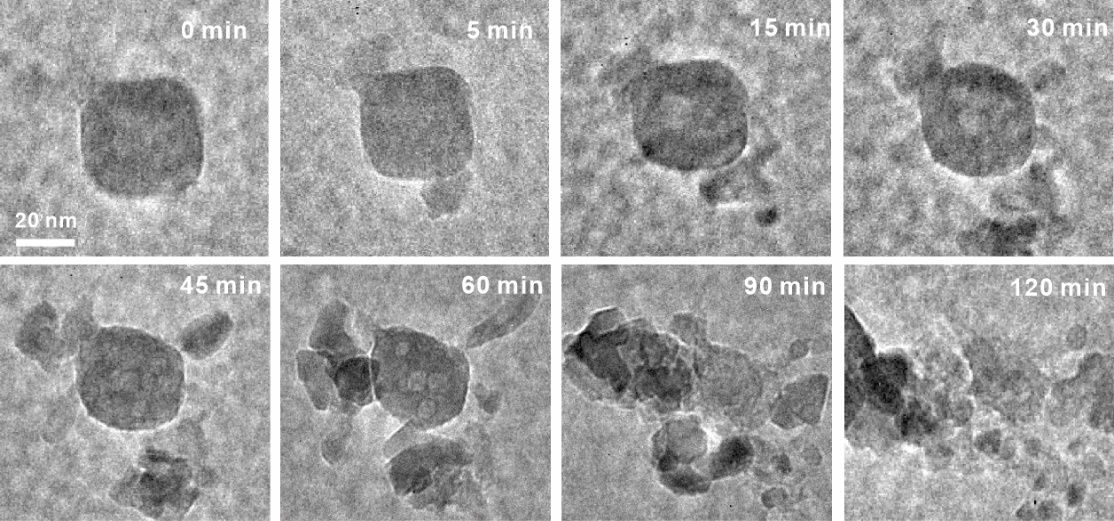


**Figure S12.** The nonclassical reduction behavior of α-Fe_2_O_3_ with dominantly exposed {012} facet.


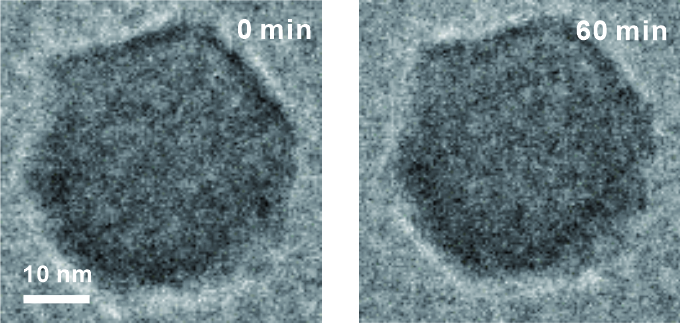


**Figure S13.** Time-resolved TEM images of the reduction of individual α-Fe_2_O_3_ nanoparticle showing no reduction behavior within a time period of 60 min at T= 650 ^o^C without the introduction of H_2_.


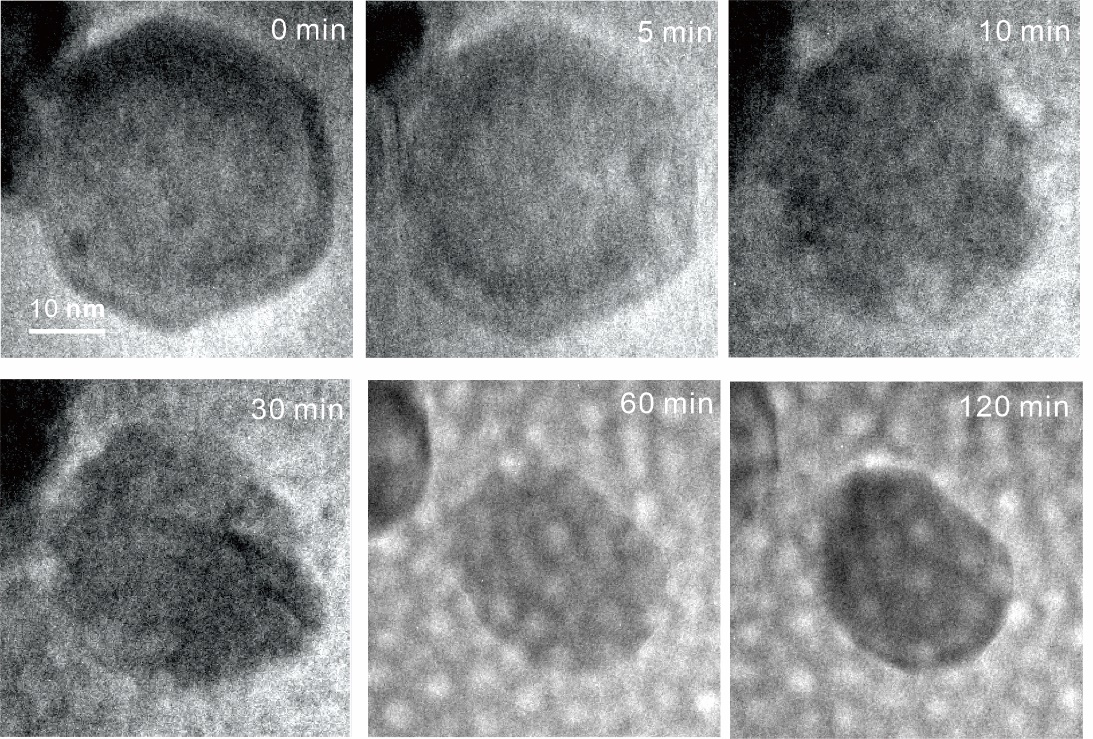


**Figure S14.** Time-resolved TEM images of the reduction of individual α-Fe_2_O_3_ nanoparticle at T= 500 ^o^C and pH_2_= 10^5^ bar showing classical interface reduction process.


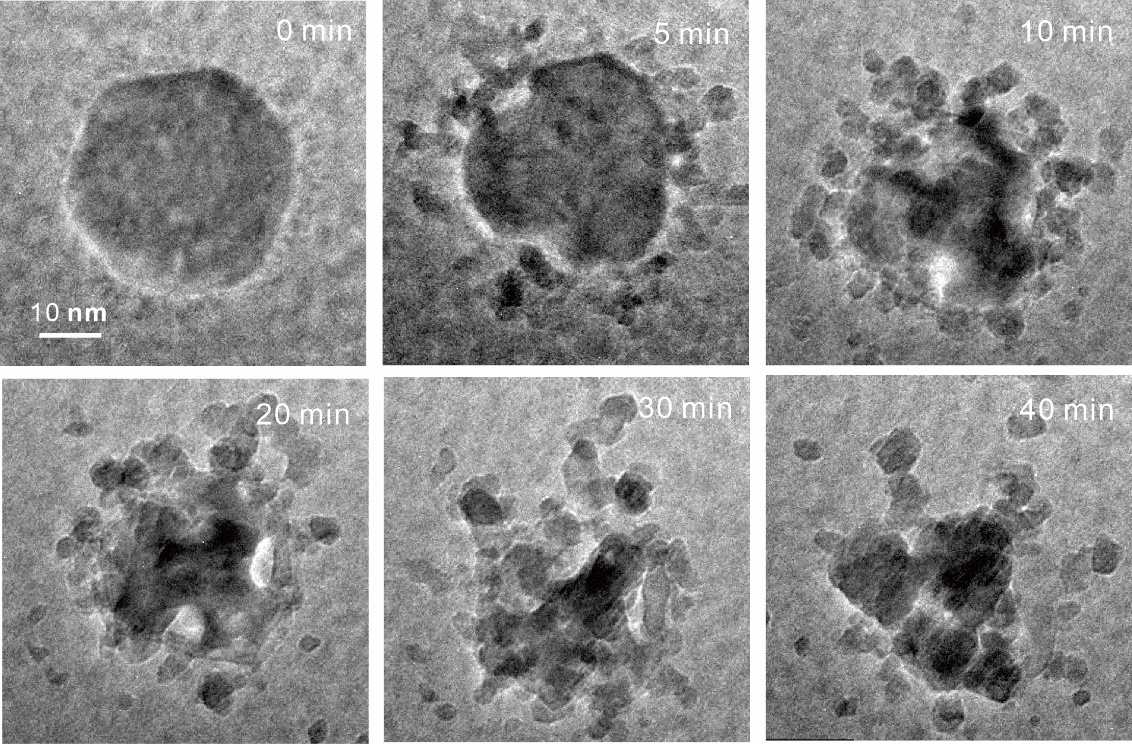


**Figure S15.** Time-resolved TEM images of the reduction of individual α-Fe_2_O_3_ nanoparticle at T= 1000 ^o^C and pH_2_= 10^5^ bar, which shows that the α-Fe_2_O_3_ nanoparticle is rapidly sintered and split.
